# Glycated haemoglobin and serum fructosamine concentrations in sick, non‐diabetic dogs receiving oral prednisolone

**DOI:** 10.1002/vetr.4843

**Published:** 2024-12-02

**Authors:** Ioannis L. Oikonomidis, Alexandra Daravigka, Jose J. Ceron, Asta Tvarijonaviciute, Froso Lambrou, Zoi Tzenetidou, Alexandros O. Konstantinidis, Nectarios Soubasis

**Affiliations:** ^1^ Department of Anatomy, Physiology and Pathology Institute of Infection, Veterinary and Ecological Studies, University of Liverpool Neston UK; ^2^ Companion Animal Clinic, School of Veterinary Medicine, Faculty of Health Sciences, Aristotle University of Thessaloniki Thessaloniki Greece; ^3^ Interdisciplinary Laboratory of Clinical Analysis, University of Murcia Murcia Spain; ^4^ Labnet Laboratories Thessaloniki Greece

**Keywords:** corticosteroids, diabetes mellitus, glucocorticoids, HbA1c, hyperglycaemia

## Abstract

**Background:**

Glucocorticoids have been associated with an increased risk of developing diabetes mellitus (DM) in dogs. Glycated haemoglobin (HbA1c) and serum fructosamine have been scarcely studied in dogs receiving glucocorticoid therapy. The aim of this study was to evaluate HbA1c and fructosamine in non‐diabetic dogs receiving oral prednisolone.

**Methods:**

This was a cross‐sectional study including non‐diabetic dogs receiving oral prednisolone for 2 weeks or more for a diverse range of diseases. Aliquots of blood samples collected for diagnostic purposes were used. HbA1c was measured using a previously validated capillary electrophoresis assay. Fructosamine was measured using the nitroblue tetrazolium assay.

**Results:**

Forty‐three adult dogs were included. The dogs received oral prednisolone (0.1‒2.3 mg/kg daily) for a median duration of 8 weeks (range: 2‒52 weeks). The median (range) glucose, fructosamine and HbA1c were 5.3 (2.6‒9.2) mmol/L, 265 (128‒388) µmol/L and 1.6% (0.8‒2.7%), respectively. Glucose increased in nine of the 43 dogs (16.2%), while fructosamine increased in eight of the 33 dogs in which it was tested (24.2%). HbA1c did not increase in any of the 40 dogs in which it was tested.

**Limitations:**

The cross‐sectional study design is a limitation, as more conclusions could be drawn in a longitudinal study.

**Conclusions:**

HbA1c does not appear to be increased in sick, non‐diabetic dogs receiving oral prednisolone for 2 weeks or more at the doses used in our study, whereas increases in serum fructosamine are not uncommon.

## INTRODUCTION

The major fraction of glycated haemoglobin (HbA1c) results from the irreversible, non‐enzymatic, insulin‐independent binding of glucose to the N‐terminal valine residue of the β‐globin chain of haemoglobin.^1^ The extent of glycosylation depends on the lifespan of erythrocytes and the average blood glucose concentration during their circulation; therefore, in dogs, HbA1c levels reflect the average blood glucose concentration over the preceding 2‒3 months.^2^ Conversely, serum fructosamine results from the irreversible, non‐enzymatic, insulin‐independent binding of glucose to plasma proteins.^1^ The extent of glycosylation depends on the average blood glucose concentration during the proteins’ lifespan. As a result, the serum fructosamine concentration reflects a dog's average blood glucose concentration over the preceding 2‒3 weeks.^1^

Serum fructosamine is widely used for monitoring and, on certain occasions, diagnosing diabetes mellitus (DM). Despite the recent interest in using HbA1c for diagnosing and monitoring DM in dogs, it has not yet been widely adopted. However, in our previous study, HbA1c was found to be 100% accurate for diagnosing DM in dogs, whereas serum fructosamine was only 84.4% accurate.^3^ In that study, we compared a diabetic population with a population of dogs with Cushing's syndrome, a population of chronically ill dogs and a population of dogs that had been undergoing treatment with glucocorticoids for at least 2 weeks. Surprisingly, more than half of the dogs undergoing glucocorticoid therapy had increased serum fructosamine, while HbA1c was not increased in any of these dogs^3^; nonetheless, the number of dogs undergoing glucocorticoid therapy was quite small in that study.

Glucocorticoids are widely used in veterinary medicine for their anti‐inflammatory and immunosuppressive effects. Nevertheless, glucocorticoids are associated with a variety of side effects.^4^ In particular, according to in vivo and in vitro studies in human medicine, glucocorticoids cause insulin resistance, which can lead to hyperglycaemia and impaired glucose tolerance, which in turn can cause exhaustion of the pancreatic β cells, resulting in the development of DM.^5^ In human medicine, glucocorticoid‐induced DM is a well‐known entity.^6^, ^7^ Glucocorticoid administration has been associated with an increased risk of developing DM in dogs in two recent studies.^8^, ^9^ In the first study, it was found that diabetic dogs had slightly more than two times greater odds of having been exposed to glucocorticoids in the preceding 6 weeks compared to non‐diabetic dogs.^9^ In the most recent study, dogs that received glucocorticoids during the 6 weeks preceding diagnosis had over four times greater odds of developing DM compared to dogs that had not received glucocorticoids.^8^

The objective of our study was to evaluate HbA1c and serum fructosamine in sick, non‐diabetic dogs that had been receiving oral prednisolone for at least 2 weeks. We hypothesised that significant increases in serum fructosamine would be observed, while HbA1c would not be increased above the reference interval (RI).

## MATERIALS AND METHODS

### Samples

All specimens used in our study were collected for diagnostic, monitoring or routine health check purposes. The present study had a cross‐sectional design and included dogs aged 1 year or older that were receiving oral prednisolone for at least 2 weeks before admission for the treatment of a diverse range of diseases. The time‐weighted mean prednisolone dose for the preceding month was calculated using the following formula: dose × duration (weeks)/4. A control group of clinically and clinicopathologically healthy dogs aged 1 year or older was also included in order to calculate the median serum albumin concentration that would be used for the calculation of corrected fructosamine. Dogs with clinical DM and serum samples with visible haemolysis were excluded from this study. The diagnosis of clinical DM was based on the presence of compatible clinical history (polyuria, polydipsia, polyphagia and weight loss) and a serum glucose concentration of 11.1 mmol/L or more.^10^ In cases where there was uncertainty over the presence of clinical signs, persistent hyperglycaemia and glycosuria were required for a diagnosis of DM.^10^ Aliquots of surplus blood from dogs that were presented to a small animal teaching hospital during a 5‐month period were used. Blood was collected via jugular venipuncture into K_3_EDTA tubes and tubes with clot activator (Deltalab). The aliquots in tubes with clot activator were allowed to clot for 20 minutes and centrifuged at 1800 *g* for 5 minutes. After centrifugation, the serum was harvested, transferred to plain tubes and stored at ‒25°C for up to 5 months before analysis.

### Measurements

A complete blood count was performed on a Cell‐Dyn 3700 (Abbott Diagnostics) within 4 hours of sampling. A previously validated capillary electrophoresis assay^11^ was used for the measurement of HbA1c within 6 hours of sampling using the EDTA whole blood samples (Capillarys 2 flex‐piercing, Sebia). Serum glucose, fructosamine, total protein and albumin were measured using an automated spectrophotometric analyser and the respective commercial kits (Olympus AU400, Beckman) using the stored serum samples. More information about the calculation of the fructosamine RI can be found in the Supporting Information. The serum globulin concentration was calculated by subtracting the albumin concentration from the total protein concentration. The following formula was used to correct serum fructosamine for serum albumin as previously described^12^: corrected fructosamine (µmol/L) = measured fructosamine × median control albumin (g/L)/measured albumin (g/L). Quality control procedures were conducted according to the manufacturer's instructions.

### Statistical methods

The Shapiro‒Wilk test was used to assess the data distribution. Depending on the data distribution, Pearson's or Spearman's correlation coefficient was used to study the correlation between two variables. All the statistical analyses were performed using the R statistical software (R Foundation for Statistical Computing).

## RESULTS

In total, 43 dogs (25 males and 18 females) undergoing prednisolone treatment were included. The diagnoses are presented in Table 1. Twenty‐four dogs belonged to 14 different pure breeds (Akita [*n* = 1], American Cocker Spaniel [*n* = 1], Beagle [*n* = 1], Bichon Frise [*n* = 1], Cocker Spaniel [*n* = 3], English Setter [*n* = 1], French Bulldog [*n* = 2], German Shepherd Dog [*n* = 2], Golder Retriever [*n* = 2], Labrador Retriever [*n* = 1], Maltese [*n* = 3], Pekingese [*n* = 2], Poodle [*n* = 1] and Shih‐tzu [*n* = 3]), while 19 were mixed‐breed dogs. The median (range) age and bodyweight were 8.0 (1.0‒16.0) years and 14.1 (2.1‒41.3) kg, respectively. A control group of 12 dogs (five male and seven female) was included in order to calculate the median serum albumin concentration that would be used in the correction formula for fructosamine. The median (range) age and bodyweight of these control dogs were 6.0 (1.5‒13.0) years and 13.0 (5.0‒31.8) kg, respectively. Their median (range) serum albumin concentration was 31.7 (30.0‒35.0) g/dL.

**TABLE 1:** Diagnoses of the 43 dogs included in the present study

Diagnosis	Number of cases
Atopy	1
Chronic otitis externa and media	1
Cutaneous lymphoma	3
Encephalitis	1
Eosinophilic gastroenteritis	2
Intestinal lymphoma	1
Heartworm disease	1
Idiopathic pericardial effusion	1
Immune‐mediated haemolytic anaemia	7
Immune‐mediated polyarthritis	10
Immune thrombocytopenia	5
Leishmaniosis	2
Lymphoplasmacytic gastroenteritis	2
Mast cell tumour	2
Meningioma	1
Myxosarcoma	1
Nasal lymphoma	1
Primary hepatic disease	1

**FIGURE 1:**
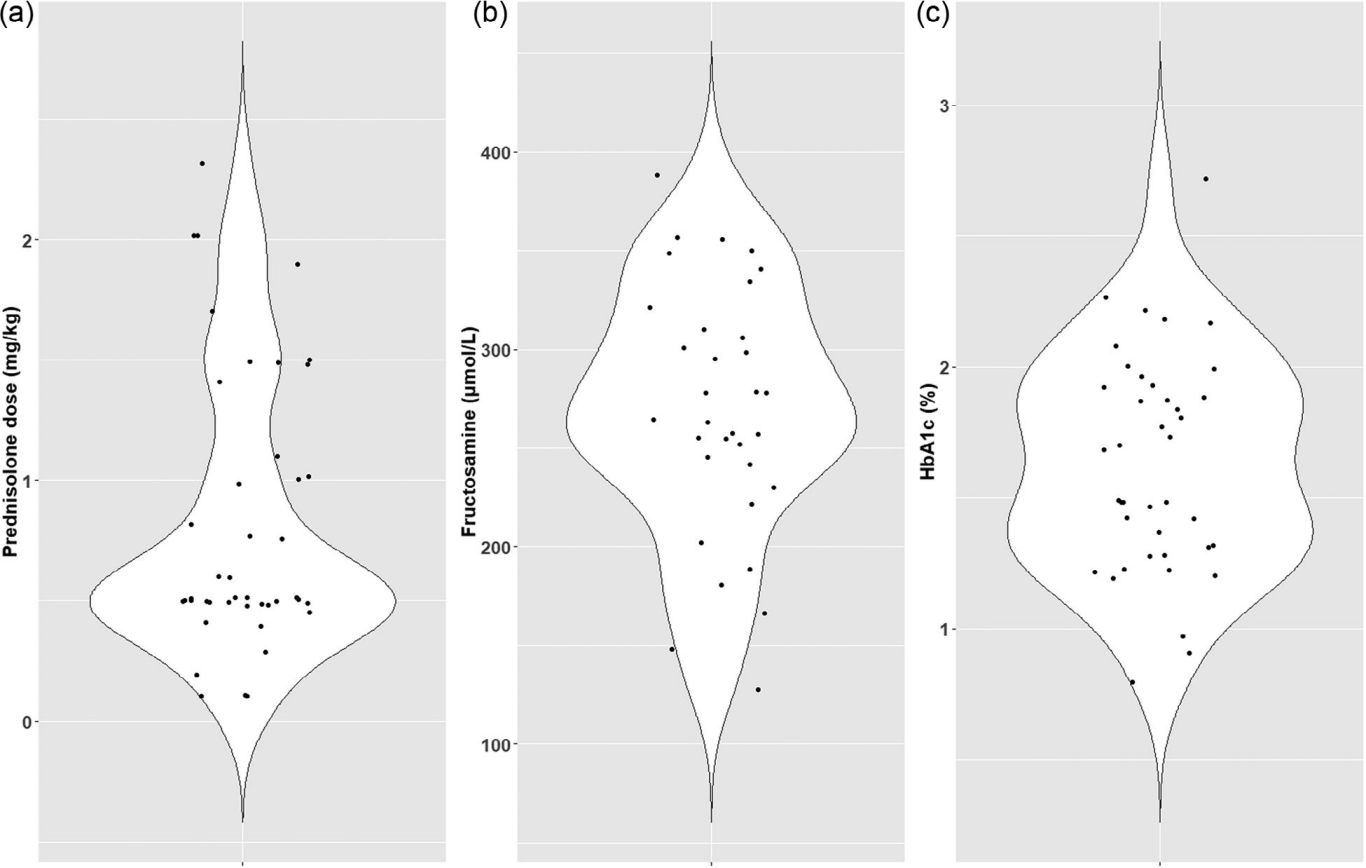
(a) The left violin plot depicts the daily doses of prednisolone during the preceding 2 weeks before blood sampling. (b) The middle violin plot depicts the serum fructosamine concentrations, and (c) the right violin plot depicts the glycated haemoglobin (HbA1c) values. Serum fructosamine was measured in 33 dogs and HbA1c was measured in 40 dogs

The dogs received oral prednisolone at doses ranging from 0.1 to 2.3 mg/kg daily for a median duration of 8 weeks (range: 2‒52 weeks). The time‐weighted mean (±standard deviation) prednisolone dose for the preceding month was 0.842 (±0.536) mg/kg daily. The descriptive statistics for selected clinicopathological analytes are presented in Tables 2 and 3. Haemoglobin was measured in all dogs, HbA1c was measured in 40 of 43 dogs and biochemical analytes, including serum fructosamine, were measured in 33 of 43 dogs. Hyperglycaemia (i.e., serum glucose >6.1 mmol/L) was observed in seven of 43 (16.2%) dogs (Figure 1). Serum fructosamine was elevated (>310 µmol/L) (Supporting Information) in eight of 33 (24.2%) dogs (Figure 1), and corrected fructosamine was increased in 10 of 33 (30.3%) dogs. All dogs had HbA1c within the RI (0.6‒2.7%)^10^ (Figure 1). The serum albumin concentration was decreased in six of 33 (18.2%) dogs, within the RI (25‒36 g/L) in 22 of 33 (66.7%) dogs and increased in five of 33 (15.1%) dogs.

**TABLE 2:** Descriptive statistics for selected normally‐distributed clinicopathological analytes from 43 non‐diabetic dogs that were receiving oral prednisolone for at least 2 weeks

Variable	N	Mean	Standard deviation	Reference interval
HbA1c (%)	40	1.6	0.4	0.6‐27
Fructosamine (µmol/L)	33	270	64	162‐310
Albumin‐corrected fructosamine (µmol/L)	33	287	52	162‐310

**TABLE 3:** Descriptive statistics for selected non‐normally‐distributed clinicopathological analytes from 43 non‐diabetic dogs that were receiving oral prednisolone for at least 2 weeks

Variable	N	Median	Range	Reference interval
Glucose (mmol/L)	43	5.3	2.6‐9.2	3.9‐6.1
Haemoglobin (g/L)	43	143	41‐267	120‐180
Total protein (g/L)	33	61	30‐76	54‐72
Albumin (g/L)	33	31	17‐37	25‐36
Globulins (g/L)	33	30	10‐50	26‐38

**FIGURE 2:**
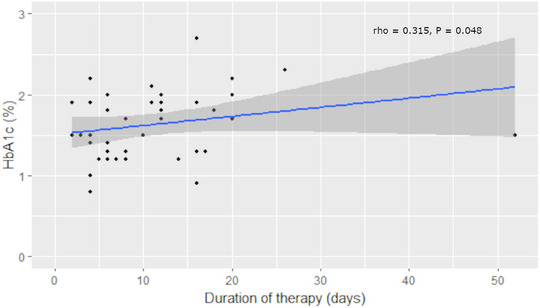
Correlation plot of glycated haemoglobin (HbA1c) and duration of prednisolone therapy. The blue line represents the regression line and the shaded areas around it represent the 95% confidence interval

HbA1c was significantly correlated with the duration of prednisolone therapy (*ρ* = 0.315, *p* = 0.048) (Figure 2), but it was not significantly correlated with haemoglobin (*ρ* = 0.280, *p* = 0.080), glucose (*ρ* = 0.110, *p* = 0.500), fructosamine (*ρ* = 0.053, *p* = 0.777) and the time‐weighted mean prednisolone dose of the preceding month (*ρ* = ‒0.080, *p* = 0.623). Fructosamine was significantly correlated with total proteins (*ρ* = 0.553, *p* < 0.001) and albumin (*ρ* = 0.608, *p* < 0.001), but it was not significantly correlated with glucose (*ρ* = 0.300, *p* = 0.090), globulins (*ρ* = 0.311, *p* = 0.079), duration of prednisolone therapy (*ρ* = ‒0.121, *p* = 0.501) and the time‐weighted mean prednisolone dose of the preceding month (*ρ* = 0.236, *p* = 0.185).

## DISCUSSION

Under the conditions of our study, HbA1c was not increased in any of the sick, non‐diabetic dogs receiving oral prednisolone. In contrast, elevated serum fructosamine was not an uncommon finding in the present study. This finding is consistent with the findings of our previous study, where none of the dogs undergoing glucocorticoid therapy had increased HbA1c.^3^ The results of the present study further support the excellent specificity of HbA1c in diagnosing DM in dogs. On the other hand, serum fructosamine concentration was elevated in one‐quarter of the dogs. Surprisingly, serum fructosamine was found to be elevated with higher frequency than serum glucose in our cohort of dogs. In our previous study, serum fructosamine was found to be 84.4% accurate in diagnosing DM in dogs.^3^ This finding indicates that serum fructosamine can be elevated under conditions other than DM, one of which is long‐term treatment with oral prednisolone. Therefore, caution is required when interpreting fructosamine results in dogs receiving glucocorticoids. This is an important observation as glucocorticoids cause insulin resistance, which can lead to overt DM in dogs.^13^, ^14^ Indeed, previous exposure to glucocorticoids was recently found to increase the risk of dogs developing DM.^8^ When the development of DM is clinically suspected in dogs receiving glucocorticoids, HbA1c might be a better test to perform than serum fructosamine.

In the light of the recent publication of the project Agreeing Language in Veterinary Endocrinology (ALIVE), our results highlight that dogs treated with glucocorticoids may meet the criteria for subclinical DM diagnosis, namely, a serum glucose concentration of between 7 and 11.1 mmol/L and an increased serum fructosamine concentration.^10^ It appears that, despite the low prevalence of DM in dogs,^9^, ^15^ glucocorticoid therapy can potentially be associated with transient subclinical DM as defined by the diagnostic criteria set out in project ALIVE.^10^

A significant, moderate, positive correlation was found between fructosamine and albumin, while no significant correlation was noted between fructosamine and glucose. This could suggest that fructosamine is primarily affected by the albumin concentration rather than the glucose concentration in non‐diabetic dogs receiving prednisolone. This is further supported by the results of our previous study, where we found that the correlation between fructosamine and albumin was substantially higher than that between fructosamine and glucose in a population of mostly normoglycaemic dogs.^3^ In an experimental study, prednisolone caused a dose‐independent increase of approximately 15‒17% in the serum albumin concentration in dogs.^16^ In our study, some of the dogs with elevated fructosamine had serum albumin concentrations near or slightly above the upper reference limit. This could suggest that an increase in serum albumin concentration might be the underlying mechanism driving increased serum fructosamine, at least in a subset of dogs. Notably, corrected fructosamine, adjusted for albumin concentration, was elevated in nearly one‐third of the dogs included in our study, indicating that serum fructosamine is also affected by other factors, which might be associated with the inherent limitations of the assay used for its measurement. Although the nitroblue tetrazolium assay is widely used, it remains poorly standardised and can be affected by changes in the ambient temperature and the presence of other reducing agents in serum, such as bilirubin and vitamins.^17^ Having said that, we should emphasise that the absence of a statistically significant correlation between fructosamine and glucose does not rule out the presence of hyperglycaemia during the preceding 2‒3 weeks.

HbA1c was significantly, positively correlated with the duration of administration of prednisolone, albeit only weakly. However, serum fructosamine was not significantly correlated with the duration of prednisolone therapy. The HbA1c levels reflect the average blood glucose concentration over the preceding 2‒3 months, while serum fructosamine concentration reflects the average blood glucose concentration over the preceding 2‒3 weeks.^1^ Given that the median duration of prednisolone administration in this study was 8 weeks, this is not an unexpected finding. Although the dose of glucocorticoids has been found to be a risk factor for the development of drug‐induced DM in people,^18^, ^19^ fructosamine and HbA1c were not significantly correlated with the time‐weighted mean prednisolone dose of the preceding month. This might be explained by the relatively low doses of prednisolone used in our cohort, as most dogs were receiving anti‐inflammatory doses of prednisolone and only five had received an immunosuppressive dose of prednisolone during the preceding 8 weeks.

However, this was a cross‐sectional study and the canine population used was not followed to assess HbA1c and fructosamine concentrations over time or detect any possible progression to overt DM. One could argue that the dogs that had increased serum fructosamine concentrations might be early diabetic, when serum fructosamine is expected to be increased but HbA1c is unaffected. However, the attending clinician for each of the cases included in our study did not have any suspicion for DM. Importantly, serum fructosamine was increased in a quarter of the dogs included in our study and it appears highly unlikely that such a high number of dogs had developed DM, as the prevalence of canine DM is generally less than 1%.^9^, ^15^ Nonetheless, a future longitudinal study might provide further information on the effect of glucocorticoids on HbA1c and serum fructosamine in dogs. Additionally, as the dogs in our study were mostly receiving anti‐inflammatory doses of prednisolone, a future study that will include dogs receiving immunosuppressive doses of prednisolone will greatly complement the current study.

In conclusion, under the conditions of our study, HbA1c did not increase above the RI in dogs receiving oral prednisolone. In contrast, an increase in serum unadjusted and albumin‐corrected fructosamine was not an uncommon finding. As such, caution is required when interpreting fructosamine results in dogs receiving prednisolone.

## AUTHOR CONTRIBUTIONS

Ioannis L. Oikonomidis conceived the idea, performed the statistical analysis and wrote the manuscript. Alexandra Daravigka, Zoi Tzenetidou and Alexandros O. Konstantinidis collected the samples and proofread the manuscript. Jose J. Ceron, Asta Tvarijonaviciute and Froso Lambrou performed the laboratory analyses and proofread the manuscript. Nectarios Soubasis supervised the research project and proofread the manuscript.

## CONFLICT OF INTEREST STATEMENT

None of the authors holds any personal or professional affiliation that may have influenced the study.

## FUNDING INFORMATION

No third‐party funding was received for the present study.

## ETHICS STATEMENT

As the blood samples used in the study were initially collected for diagnostic, monitoring or routine health check purposes, the institutional ethics committee deemed that ethical approval was not required. Owner consent was obtained for the use of these surplus blood samples for research purposes.

## Supporting information



Supporting Information

## Data Availability

Data are available from the corresponding author upon reasonable request.
