# Supplementary material for: Glycated haemoglobin and serum fructosamine concentrations in sick, non‐diabetic dogs receiving oral prednisolone
Source: Vet Rec. 2024 Dec 2;196(2):e4843. doi: 10.1002/vetr.4843 (PMC11740410; doi:10.1002/vetr.4843)
Supplement: Supplementary file 1 — Supporting Information [file VETR-196-e4843-s001.docx]

**Fructosamine reference interval study**

The reference population was composed of 54 dogs with a mean (±SD) age of 2.8 (±2.5) years. All dogs were clinically healthy with unremarkable complete blood count and standard biochemistry profile. The same assay and biochemistry analyser that were used in the present study were used for the measurement of serum fructosamine concentration. Specifically, a colorimetric assay (BioSystems, 11046; BioSystems S.A, Spain) adapted to an automated biochemistry analyser (Olympus AU400, Olympus Diagnostica GmbH1, Hamburg, Germany) was used to measure fructosamine. The reference interval was calculated as mean ± 2SD, according to a previously published study on the reference interval of feline fructosamine (Thoresen et al., 1995). All the measurements and the statistical analyses were performed in the Interdisciplinary Laboratory of Clinical Analysis, University of Murcia, Spain.

**Reference**

Thoresen, S.I. and Bredal, W.P. Determination of a reference range for fructosamine in feline serum samples. Veterinary Research Communications. 1995;19(5):353-361.
